# Draft Genome Sequence of *Streptomyces niveus* NCIMB 11891, Producer of the Aminocoumarin Antibiotic Novobiocin

**DOI:** 10.1128/genomeA.01146-13

**Published:** 2014-01-09

**Authors:** Katrin Flinspach, Christian Rückert, Jörn Kalinowski, Lutz Heide, Alexander Kristian Apel

**Affiliations:** Pharmaceutical Institute, Tübingen University, Tübingen, Germanya; Technology Platform Genomics, CeBiTec, Bielefeld University, Bielefeld, Germanyb

## Abstract

*Streptomyces niveus* NCIMB 11891 is the producer of the gyrase inhibitor novobiocin, which belongs to the aminocoumarin class of antibiotics. The genome sequence of this strain was found to contain, besides the gene cluster for novobiocin, a putative gene cluster for the macrolactam antibiotic BE-14106 and further secondary metabolite gene clusters.

## GENOME ANNOUNCEMENT

The aminocoumarin antibiotic novobiocin is a potent gyrase inhibitor binding to the GyrB subunit of bacterial gyrase (1). It has been in clinical use under the name albamycin and is still used in veterinary medicine. Novobiocin was discovered from a soil bacterium that was originally termed *Streptomyces spheroides* (2), which was later reclassified as *Streptomyces caeruleus* (3) and eventually as *Streptomyces niveus* NCIMB 11891 (4). An independent novobiocin-producing isolate was termed *S. niveus* NCIMB 9219 (5).

In the draft genome sequence of *S. niveus* NCIMB 11891, we identified the previously published novobiocin cluster (GenBank accession no. AF170880) (6). However, we found 543 bp of the 28,076-kb gene cluster to be different from that of the novobiocin cluster, with most of the differences representing conservative mutations. This prompted us to reorder the strains *S. niveus* DSM 40292 (= NCIMB 11891) and *S. niveus* DSM 40088 (= NCIMB 9219) from the DSMZ strain collection (Braunschweig, Germany). PCR amplification and sequencing of different regions of the novobiocin cluster from both strains unequivocally confirmed that the previously published sequence had been obtained from *S. niveus* type strain NCIMB 9219, not from strain NCIMB 11891. GenBank accession no. AF170880 was corrected accordingly.

Furthermore, a gene cluster was identified with 97% identity on the nucleotide level with a sequence (accession no. FJ872523.1) from *Streptomyces* sp. strain DSM 21069, a gene cluster for the macrolactam antibiotic BE-14106 (7). *Streptomyces* sp. DSM 21069 is closely related to *S. niveus*, showing 99% 16S rRNA identity.

The program antiSMASH (8) revealed 27 further gene clusters for the biosynthesis of secondary metabolites (1 melanin, 2 siderophore, 5 terpene, 1 mixed terpene/polyketide synthase [T1-PKS], 6 PKS [2 T1-PKS, 3 T3-PKS, 1 T4-PKS], 1 mixed butyrolactone/PKS [T1-PKS], 1 butyrolactone, 1 ectoine, 2 bacteriocin, 3 nonribosomal peptide synthetase [NRPS], 1 nucleoside, 1 lantipeptide, 1 thiopeptide-lantipeptide, and 1 unspecified cluster).

The genomic sequence of *S. niveus* was obtained by assembly of two data sets, which were generated by paired-end and whole-genome shotgun pyrosequencing strategies (9) utilizing the Newbler software (version 2.5.3). Overall, 725,706 reads were assembled to an 8,702,051-nucleotide draft at 23.0-fold coverage. The resulting draft genome sequence consists of 509 contigs in total (321 contigs >500 bases) in 10 scaffolds, with an overall G+C content of 70.74%. Manual *in silico* assembly revealed the presence of four linear plasmids, pSniv1 to pSniv4, with sizes between 123.3 kb and 15.8 kb. The final draft sequence consists of five scaffolds, one for the linear chromosome and one for each of the four plasmids, consisting of 309 contigs/8.51 Mbp (chromosome), 3 contigs/123.3 kbp (pSniv1), 2 contigs/53.4 kbp (pSniv2), 3 contigs/19.4 kbp (pSniv3), and 1 contig/15.8 kbp (pSniv4) The assembled contigs were annotated with the PGAAP pipeline (10), resulting in the annotation of 7,799 coding sequences (CDSs). Furthermore, we identified 7 rRNA operons and 66 tRNA loci.

The *S. niveus* genome sequence will allow further experiments to be carried out regarding the regulation of novobiocin biosynthesis and for new gene clusters and bioactive compounds to be mined.

### Nucleotide sequence accession numbers.

This whole-genome shotgun sequence has been deposited in DDBJ/EMBL/GenBank under accession no. AWQW00000000. The version described in this paper is version AWQW01000000.
